# Connexin43 expression in bone marrow derived cells contributes to the electrophysiological properties of cardiac scar tissue

**DOI:** 10.1038/s41598-020-59449-7

**Published:** 2020-02-13

**Authors:** Carolina Vasquez, Valeria Mezzano, Newman Kessler, Freja Swardh, Desiree Ernestad, Vanessa M. Mahoney, John Hanna, Gregory E. Morley

**Affiliations:** Leon H. Charney Division of Cardiology, Department of Medicine, New York University School of Medicine, New York, NY 10016 USA

**Keywords:** Physiology, Cardiology

## Abstract

Cardiac pathologies associated with arrhythmic activity are often accompanied by inflammation. The contribution of inflammatory cells to the electrophysiological properties of injured myocardium is unknown. Myocardial scar cell types and intercellular contacts were analyzed using a three-dimensional reconstruction from serial blockface scanning electron microscopy data. Three distinct cell populations were identified: inflammatory, fibroblastic and endocardial cells. While individual fibroblastic cells interface with a greater number of cells, inflammatory cells have the largest contact area suggesting a role in establishing intercellular electrical connections in scar tissue. Optical mapping was used to study the electrophysiological properties of scars in fetal liver chimeric mice generated using connexin43 knockout donors (bmpKO). Voltage changes were elicited in response to applied current pulses. Isopotential maps showed a steeper pattern of decay with distance from the electrode in scars compared with uninjured regions, suggesting reduced electrical coupling. The tissue decay constant, defined as the distance voltage reaches 37% of the amplitude at the edge of the scar, was 0.48 ± 0.04 mm (n = 11) in the scar of the bmpCTL group and decreased 37.5% in the bmpKO group (n = 10). Together these data demonstrate inflammatory cells significantly contribute to scar electrophysiology through coupling mediated at least partially by connexin43 expression.

## Introduction

Many cardiac pathologies associated with arrhythmic activity are also associated with a complex immune response^1^. In recent years there has been increasing interest in identifying the specific inflammatory cell populations that are involved in cardiac injury and repair, determining how specific cell types change temporally and spatially, and elucidating the complex interactions between inflammatory cells and other cardiac cell populations with the ultimate goal of developing viable immunotherapy approaches for the treatment of cardiac disease^2–10^.

In myocardial infarction acute ischemic injury mobilizes a diverse group of cells ultimately leading to a large scale recruitment of neutrophils and monocytes^11,12^. These cells mostly originate from hematopoietic and stem cells from the bone marrow. Neutrophils numbers decrease significantly during the first week after injury and are almost absent by day 7, while monocytes continue to increase in numbers and differentiate into cardiac macrophages. Cardiac macrophages contribute to different aspects of cardiac function and show highly dynamic gene expression patterns and tissue composition^13^. The contribution of macrophages to the electrophysiological function of the normal heart is beginning to be elucidated^14^. However, the direct contribution of macrophages to the electrophysiological properties of the injured myocardium remains unknown.

Macrophages could contribute to arrhythmias via different mechanisms including cytokine release that directly affects myocyte function, crosstalk with fibroblasts leading to increased fibrosis, or by directly regulating electrical activity through intercellular coupling. Earlier studies from our laboratory utilized a cardiac injury model and a fibroblast-specific protein-1 (Fsp1) Cre mouse to target connexin43 (Cx43) expression in cardiac non-myocytes^15^. Fsp1 is expressed in a subset of fibroblast cells and myeloid cells. This study demonstrated the presence of functional electrical connections between uninjured myocardial tissue and non-myocytes in scar tissue. This study further showed that this communication is at least partially mediated by Cx43.

A key aspect in understanding arrhythmic activity is understanding the structural and cellular substrate that supports it. The organization of non-myocyte cell populations in scar tissue remains poorly characterized, in particular it is not known what cells establish intercellular contacts and to what extent. This void in knowledge is partly due to technical spatial resolution limitations, as well as long held views that minimized or even negated the contribution of the cardiac non-myocyte cell populations to the electrical function of the heart. Here, we performed detailed structural analyses to characterize intercellular contacts in cardiac scar tissue and identify cellular networks that could potentially support the spread of electrical activation. Furthermore we utilized a bone marrow replacement model with Cx43 knock out donor mice to study how intercellular coupling through inflammatory cells regulate the electrical properties of the scar. The findings of this study provide important new information on the role of inflammatory cells in establishing the electrophysiological properties of scar tissue 30 days after cardiac injury.

## Methods

### Experimental animals and study design

All methods were carried out in accordance with relevant guidelines and regulations. All procedures were approved by the Institutional Animal Care and Use Committee of the New York University School of Medicine (Protocol 160408-01) and complied with the standards for the care and use of animal subjects as stated in the Guide for the Care and Use of Laboratory Animals. This study was performed using 2–4 month old male C57BL/6 mice for immunofluorescence, Scanning Electron Microscopy, Transmission Electron Microscopy and Immunogold studies, 2–4 month old male fetal liver chimeric mice, and mouse embryos obtained from Cx43 heterozygote pregnant mice. C57BL/6 mice were purchased from Charles River Laboratories.

### Generation of fetal liver chimeric mice

Bone marrow progenitor cells (bmp) were obtained from fetal livers. To generate fetal liver donors, mCherry reporter transgenic mice (B6(Gg)-Tyrc-2JTg(UBC-mCherry)1Phbs/J31, Jackson Laboratories #17614) were crossed with Cx43 heterozygote knock out mice (B6;129-Gja1tm1Kdr/J32, Jackson Laboratories #2201). Fetal liver chimeric mice were generated as previously described^16^. Briefly, recipient C57BL/6 mice underwent total body gamma irradiation at a final dose of 1100 cGy (550 cGy delivered twice separated by 4 hours). Donor fetal livers were obtained from E17-E19 fetuses. Fetuses were individually genotyped and the fetal liver was mechanically dissociated and suspended in 500–800 μl RPMI1640 (Gibco). Each liver was used to transplant four myeloablated mice (~1 × 10^6^ nucleated cells each). Cells were delivered by retro-orbital injection. Quantification of bone marrow derived cells in the scars was performed by counting mCherry expressing cells in sections from injured hearts. Fetal liver chimeras were divided into groups according to the fetal donor genotype.

### Cardiac injury model

Myocardial scars were generated one month after myeloablation and transplantation by thermal ablation of the right ventricular (RV) free wall as previously described^15,17^. Briefly, chimeric mice were anaesthetized with inhaled isoflurane. A thin bipolar electrode (FHC Inc., Bowdoin, ME, USA) was introduced through an abdominal incision and advanced towards the thoracic cavity. The electrode was inserted through the diaphragm to make contact with the RV free wall. Pulsed direct current (8.0 mA, 100 ms interval, 30 ms pulse duration) was applied for 90 seconds. The electrode was removed and the abdominal wall sutured. Mice were allowed to recover and returned to the vivarium. All subsequent experiments were performed 30 days after the injury procedure.

### Transmission Electron Microscopy (TEM)

Samples for TEM were obtained from C57Bl/6 mice 30 days after the RV injury procedure. Hearts were perfused with 15 ml PBS followed by 10 ml 4% paraformaldehyde. The RV free wall was excised and further fixed in 2.5% glutaraldehyde and 2% paraformaldehyde in 0.1 M phosphate buffer (pH 7.4) for 2 hours followed by 1% osmium tetroxide for 1.5 hours. The samples were processed in a standard manner and embedded in EMbed 812 (Electron Microscopy Sciences, Hatfield, PA). Semi-thin sections were cut at 1 mm and stained with 1% toluidine blue to evaluate the quality of preservation. Ultrathin sections were cut at 60 nm, and stained with uranyl acetate and lead citrate using standard methods. Stained grids were examined under electron microscope (Philips CM-12) and photographed with a digital camera (Gatan; 4k × 2.7k) to identify regions for Serial Blockface Scanning Electron Microscopy.

Samples for immuno-electron microscopy were obtained from one C57Bl/6 and one bmpCTL mice 30 days after the RV injury procedure. Following perfusion and dissection, scars samples were fixed in freshly made 3% paraformaldehyde in 0.1 M phosphate buffer containing 0.1% glutaraldehyde and 4% sucrose (pH 7.2). After washing and dehydration, the tissues were embedded in LR White (Electron Microscopy Sciences, Hatfield, PA), and polymerized at −52 to −55 °C. 100 nm thin sections were cut and mounted on formvar-carbon coated nickel grids. After incubation with primary antibodies (AB1728, Millipore) at 4 °C overnight, 15 nm protein A gold (Cell Microscopy Center, University Medical Center Utrecht, 35584 CX Utrecht, The Netherlands) was applied and stained with uranyl acetate and lead citrate by standard methods.

All stained grids were examined either under a Philips CM-12 electron microscope (FEI; Eindhoven, The Netherlands) and photographed with a Gatan (4k × 2.7k) digital camera, or Talos120C transmission electron microscope (Thermo Fisher Scientific, Hillsboro, OR) with Gatan (4k × 4k) OneView Camera (Gatan, Inc., Pleasanton, CA).

### Serial Blockface Scanning Electron Microscopy (SBF-SEM)

#### Specimen preparation

Samples for SBF-SEM were obtained from C57Bl/6 mice 30 days after RV injury. Hearts were perfused *in situ* with PBS followed by 4% paraformaldehyde. The heart was excised and the RV free wall was dissected and further fixed overnight in a glutaraldehyde/PFA solution (2.5%/2%, respectively). Heavy metal staining was performed by incubating the sample for 2 hours in 2% osmium tetroxide with 1.5% potassium ferrocyanide, followed by 20 minute incubation in 0.01% thiocarbohydrazide and 30 min incubation in 2% osmium tetroxide. The sample was then incubated overnight at 4 °C in 1% uranyl acetate in H_2_O followed by a 30 min incubation in Walton’s lead aspartate pH 5.5 (0.066 g lead nitrate in 10 ml aspartic acid) at 60 °C. Following staining the sample was embedded in Durcupan. The region of interest consisting of an area at the center of the RV injury containing only scar tissue was chosen through examination of ultrathin sections through TEM before serial blockface acquisition. SBF-SEM images were acquired with a 3 View system (Gatan, Abindgon, UK) retrofitted to Zeiss GEMINI 300 VP field-emission scanning electron microscope at Zeiss Microscopy Labs (NY, USA). The dataset of 384 slices was obtained from a block of the following dimensions: Width: 75.8 μm; height: 37.9 μm; depth: 30.8 μm. Pixel size for each image was 6.3 × 6.3 nm. Slices were sectioned at 80 nm thickness. Data alignment was performed at Zeiss Microscopy laboratories.

#### Data processing

Three-dimensional reconstruction of SBF-SEM data was used to morphologically identify cell types and intercellular contacts in a wild type mouse RV scar. Tiff format files were manually segmented using VASTlite^18^ and mesh models were generated with VASTtools in MATLAB® (Mathworks®, USA). Individual cell surface areas and volumes were quantified using VASTtools. Mesh models were imported into Blender (Blender.org, the Netherlands) using the NeuroMorph^19^ plugin and intercellular contact areas were quantified using NeuroMorph. Visual representation of the data was performed using Blender, VAST and Photoshop® (Adobe®, USA). Animations were rendered in Blender.

### Immunofluorescence for Connexin43 and pan-Cadherin

Immunostaining was performed on LR-white 60 nm sections of cardiac scar tissue using anti-Connexin43 and anti-pan Cadherin antibodies (Table 1) followed by Anti-Rabbit Alexa 647 and Anti-Mouse Alexa 488. Nucleic acids were labeled with DAPI and slides were mounted using Fluoromount (DAKO). Imaging was performed using Applied Precision Personal DV Live-Cell Imaging System with an Olympus PlanApo N 60×/1.42 Oil objective. All images were acquired at pixel size of 0.107 × 0.107 × 0.200 μm (X-Y-Z) at four focal planes and deconvolved using API’s Resolved3D software (version 6.0.0 Release 9).

**Table 1:** Immunophenotyping antibodies (AB).

Target antigen	Company	Host	Clone	Catalog #	Dilution
Secondary AB	Company	Host	Clone	Catalog #	Dilution
Connexin43	Millipore	Rabbit		AB1728	1:500
Pan-Cadherin	Sigma	Mouse		C1821	1:500
F4/80	CST	Rabbit	D2S9R	70076S	1:200
mCherry	Sicgen	Goat	polyclonal	AB0040–200	1:200
anti-Rabbit IgG (H + L) Alexa Fluor 647	Invitrogen	Donkey	polyclonal	A32795	1:150
anti-Mouse Alexa Fluor 488	Invitrogen	Goat	polyclonal	A32723	1:150
Rabbit-on-Rodent HRP-Polymer	Biocare Medical	Not applicable		RMR 622	undiluted
Goat-on-Rodent HRP-Polymer	Biocare Medical	Not applicable		GHP516	undiluted

### Immunophenotyping

Five-micron sections of paraffin embedded tissue were stained with Akoya Biosciences® Opal™ multiplex automation kit reagents unless stated otherwise. Automated staining was performed on Leica BondRX® autostainer. The protocol was performed according to manufacturers’ instructions with the antibodies listed in Table 1. Briefly, all slides underwent sequential epitope retrieval with Leica Biosystems epitope retrieval 2 solution (EDTA based, pH9, Cat. AR9640), primary and secondary antibody incubation and tyramide signal amplification with Opal® fluorophores Op620 and Op520. Primary and secondary antibodies were removed during epitope retrieval steps while fluorophores remain covalently attached to the tissue.

Semi-automated image acquisition was performed on a Vectra® 3.0 multispectral imaging system. After whole slide scanning at 10X the tissue was manually outlined to select fields for multispectral imaging at 20X. InForm® (Akoya Biosciences™) was used for analysis of multispectral images^20–25^. A proprietary training algorithm was first used to segment images into three categories: Muscle, Scar and No Tissue. The following parameters were used for training: Fluorescent signal used (‘Components for Training’): DAPI, Opal520, Opal620 and Autofluorescence. Pattern scale: small. Segmentation Options: Segmentation Resolution: Fine. Post-processing parameters for the ‘Muscle’ category only: Trim edges by 5 pixels and Minimum segment size 1000 pixels. The ‘No Tissue’ category was excluded from further analysis. Tissue segmentations were reviewed for different samples during training iterations. Following tissue classification cells were segmented based on DAPI signal (labeling double stranded DNA present in the nuclei). Cell segmentation parameters were set as follows: Fluorescent signals used (‘Components’): DAPI (‘Nucleus, relative intensity’ 0.5), Autofluorescence (‘membrane signal’), Opal520 (‘membrane signal assist nuclear splitting’); Nuclear splitting sensitivity: 0.9; minimum nuclear size (pixels): 30, Assisting staining: ‘continuous with sharp edges’; Assisting Component Splitting Sensitivity: 1.20, Minimum nuclear size: 19 (pixels), Fill Nuclear Holes Smaller Than: 10; Membrane Search Distance: 5.0, Membrane Signal Threshold: 1.20. Cells were phenotyped after segmentation using inForm’s trainable algorithm. All cells recognized by nuclear stain were divided into four phenotypic categories: “F4/80(+)”, “mCherry(+)”, “F4/80(+)/mCherry(+)” and “other” which included all of the cells that did not fall into any of the previous categories. Phenotypes were reviewed for different samples during training iterations. Data wrangling and calculations were performed in RStudio software [RStudio Team (2015). RStudio: Integrated Development for R. RStudio, Inc., Boston, MA URL http://www.rstudio.com/].

### Optical mapping

Electrical coupling between the myocardium and scar was evaluated using high resolution optical mapping. Briefly, animals were administered heparin (heparin sodium, 0.5 U/g IP) and euthanized by CO_2_ inhalation followed by cervical dislocation. Hearts were excised, cannulated and Langendorff perfused using a modified Tyrode’s solution as previously described^15^. The excitation-contraction uncoupler blebbistatin (13.75 μM/L) was added to the perfusate, and the hearts were stained with the voltage-sensitive dye di-4-ANEPPS. Voltage dependent signals were recorded as previously described using a SciMedia MiCAM ULTIMA camera. Images were acquired at 1,000 frames per second with a spatial resolution of 92.8 μm/pixel.

Current pulses (5 mA, 100 ms duration, 200 ms basic cycle length) were delivered to the uninjured myocardium adjacent to the scar using a fire polished suction electrode as previously described^15^. Flecainide (20 μg/ml) was added to the perfusate to suppress action potential generation during imaging. Maximal membrane voltage amplitude in response to the passive spread of current was evaluated within the scar and in an uninjured area proximal to the scar. The uninjured area was selected to be of the same size and located at the same distance from the electrode as the scar. The spatial changes in membrane potential amplitude in response to the applied current for scar and uninjured areas were analyzed and compared between groups.

### Statistical analysis

Results are presented as mean ± SEM. Single factor ANOVA and independent *t-*tests were used to statistically compare average maximal voltage amplitude and decay constants between groups. Exponential least squares fitting was used to calculate the decay constant of scar and uninjured tissue. A *p*-value less than 0.05 was considered to be statistically significant.

## Results

**Figure 1:**
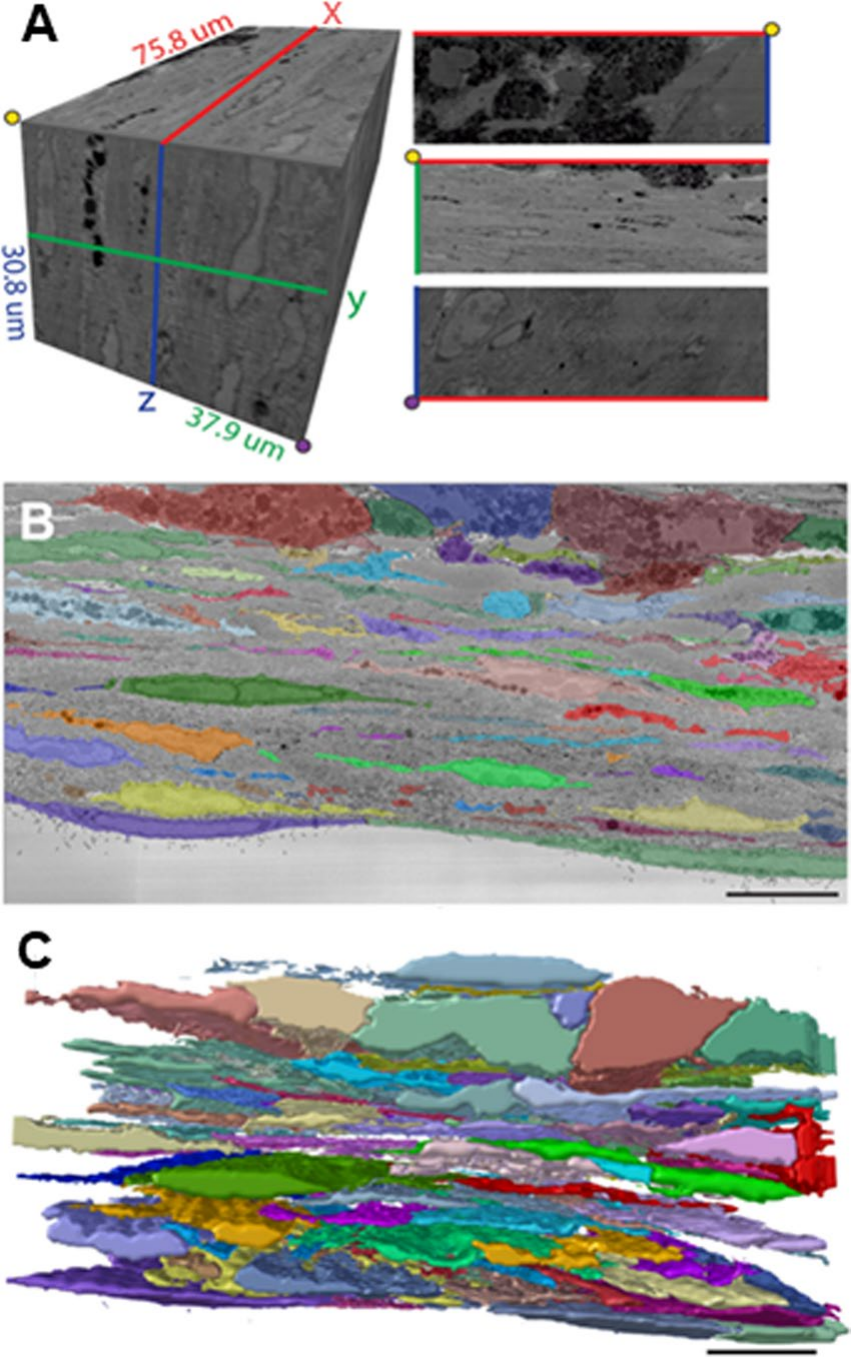
Scanning electron microscopy of myocardial scar. (**A**) Digital reconstruction of serial blockface scanning electron microscopy image dataset showing different planes of view and dimensions. (**B**) Representative segmentation of one slice showing individual cells in different colors. Bar = 10 µm. (**C**) Three-dimensional rendering of the segmented cells present in the scar. Bar = 10 µm.

The arrhythmogenic potential of scars that are highly populated with non-excitable cells is dependent on their ability to form a continuous network that is coupled to the surrounding myocardium^26,27^. However, neither the physical substrate that is responsible for coupling between scar tissue and cardiac myocytes^15,28^ nor the cellular network present within this tissue have been fully described. We have characterized a non-myocyte cellular network in a 30-day old RV scar through detailed analyses at ultrastructural resolution using SBF-SEM. Supplementary Video 1 shows an animation of a representative slice of the SBF-SEM stack. The video shows the magnitude of the x-y area that was analyzed and zooms-in to reveal the highest working magnification. The digitally reconstructed dataset had a total volume of 88,482.86 μm^3^. Figure 1A displays a three-dimensional digital reconstruction of the data set showing the different planes of view and dimensions of the stack. Cellular membranes were identified, and individual cells were segmented by following the cell membranes at high resolution (12 nm in X-Y) (Fig. 1B,C). An animation of a subset of the three-dimensional cell models together with the EM stack from which they originated is shown in Supplementary Video 2. The cell membrane segmentation yielded a total of 72 cells of which 46 contained nuclei that was visible within the dataset. Interspersed cellular processes throughout the scar divide the extracellular matrix into smaller fragments and provide physical contacts between cells.

**Figure 2:**
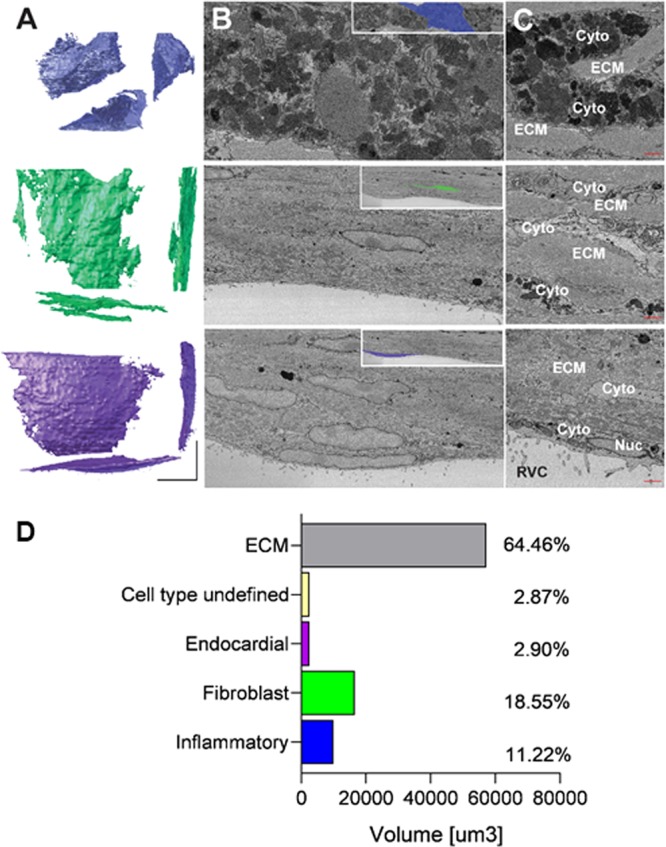
Cell volume and types of cells present in the scar. **(A**) Representative three-dimensional projection of each cell type present in scars. Each cell is rendered from X-Z (top left), Y-Z (top right) and X-Y (bottom) perspective. Inflammatory (top); fibroblastic (middle) and endocardial (lower). (**B**) SEM micrographs of the cell types in panel A. Left panels show the segmented cell at low magnification; insets show the segmentation. (**C**) Representative micrographs of ultrastructural characteristics of each cell type. Red bars = 1 µm. ECM = extracellular matrix, Cyt = cytoplasm, Nuc = nucleus, RVC = right ventricular cavity. (**D**) Volume distribution quantified for individual cell populations. Cell segments too small to properly identify as belonging to a specific population were labeled as “Undefined”.

Three distinct cell types were identified based on cellular morphology and traditional electron microscopy criteria: inflammatory, fibroblastic and endocardial cells. Figure 2A,B shows representative three-dimensional models of each cell type and SEM images exemplifying the ultrastructural characteristics of each group. Inflammatory cells were characterized by a globular morphology and the presence of abundant cytoplasmic vacuoles of varying electron density. The nuclei were more electron dense than the neighboring cells and the nucleolus showed less contrast than that of fibroblasts. These features indicate this population is primarily composed of macrophages. Fibroblastic cells were characterized by a sheet-like morphology, less electron dense cytoplasm with rough sarcoplasmic reticulum, lack of basement membrane and abundant cellular extensions and processes. Endocardial epithelial cells were identified based on location within the scar, having a planar morphology and villi facing the ventricular cavity. Analysis of individual cell volumes indicated that approximately 35.2% of the scar is occupied by cells. Figure 2D shows a breakdown of the volumes of each cell type within the scar.

**Figure 3:**
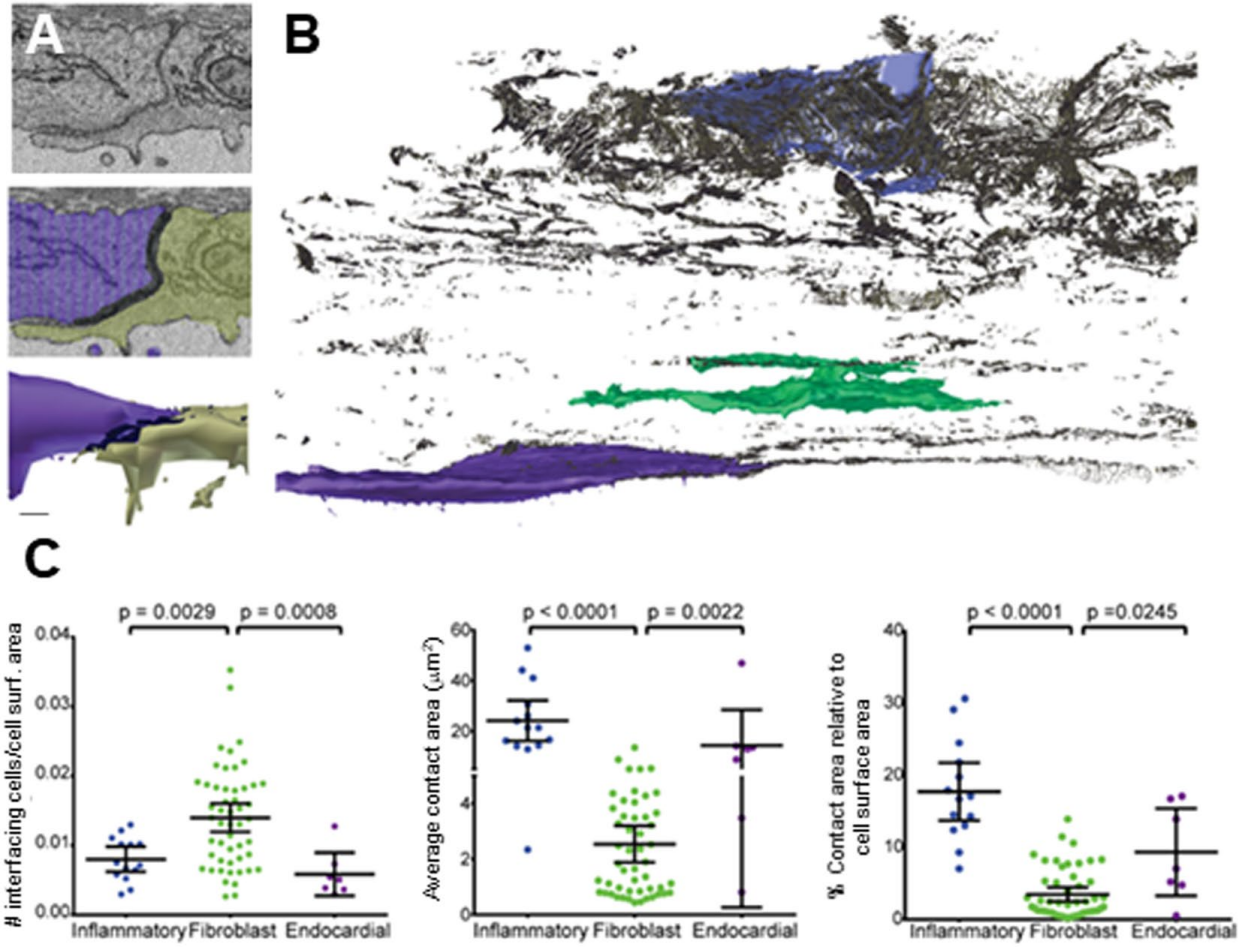
Cell and network characteristics. (**A**) Segmentation of intercellular contacts. EM micrograph of an intercellular contact between two endocardial cells (top), segmentation of the two cells and the intercellular contact, shown in black (middle). Three-dimensional model of the two cells showing the cell junctional interface (bottom). (**B**) Three-dimensional model of intercellular contacts (black areas) present in the SEM volume. Representative individual inflammatory, fibroblastic and endocardial cells are shown in their anatomical location. (**C**) Plots showing quantification of interfacing cells and cell contacts.

Individual intercellular contacts in each cell were segmented to measure the area of apposition between cells. For the purpose of segmentation intercellular contacts were defined as areas where the extracellular space between plasma membranes of two adjacent cells was unidentifiable (Fig. 3A). The segmentation and three-dimensional modelling also showed cell networks that could potentially support electrical propagation in the scar (Fig. 3B). Intermembrane distances between non-myocyte cells were measured at higher resolution to assess whether the segmented regions in the SBF-SEM stack were consistent with intercellular junction structures. No significant differences were found between non-myocyte intercellular distances at contact areas and intermembrane distances of adherens junctions in surrounding myocytes (see Supplementary Figure 1). Analysis of the three-dimensional intercellular contact models showed that on average each individual fibroblastic cell interfaces with the largest number of cells. However macrophages have appositional areas, per intercellular contact and relative to total cell surface area, which are an order of magnitude larger than appositional areas in fibroblasts (Fig. 3C). Consistent with previous studies^15,29^ and our own immunofluorescence and immunogold-TEM data obtained in these cohorts (Supplementary Figs. 2 and 3), Cx43 immunoreactivity is reproducibly found in cardiac scar regions devoid of myocytes. These findings suggest macrophages could significantly contribute to intercellular electrical communication in the scar.

The structural data suggest a prominent role for macrophages in the establishment of electrical communication between the uninjured myocardium and scar areas of the RV injury model. A fraction of the macrophage population that resides in scar tissue is derived from the bone marrow^30^. To test whether the homing of bone marrow derived cells, and more specifically macrophages to the injured area is affected by changes in Cx43 expression, mice underwent radiation-induced myeloablation and subsequent transplantation with bone marrow progenitors obtained from fetal donor cells constitutively expressing mCherry protein. Fetal liver chimeras were divided into three groups according to the fetal donor genotype: Cx43 wild type fetal liver donors (bmpCTL), Cx43 heterozygote fetal liver donors (bmpHET), and Cx43 knock out fetal liver donors (bmpKO). RV injury was produced thirty 30 days post transplantation and hearts were studied 30 days after injury.

**Figure 4:**
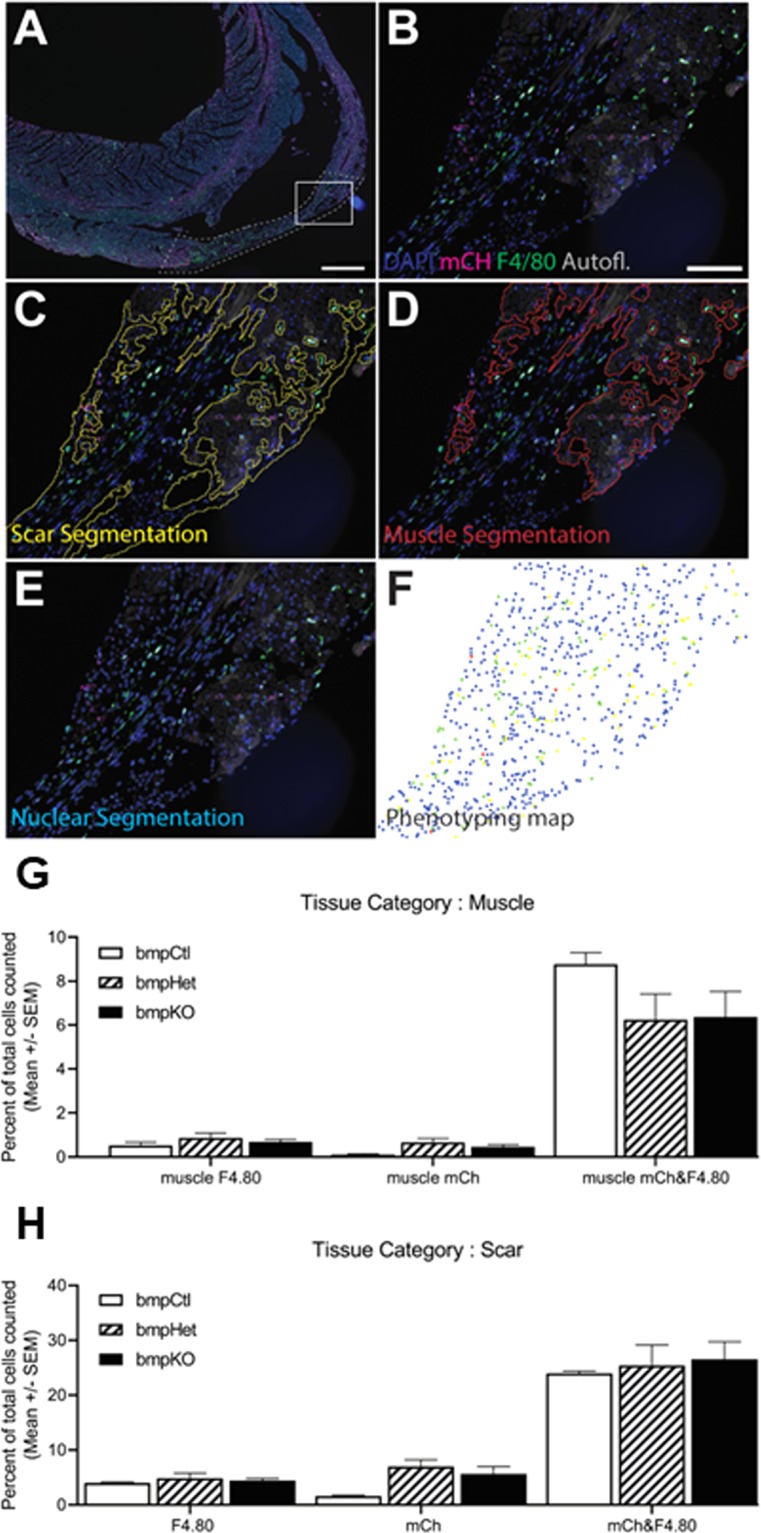
Macrophage and bone marrow derived cell numbers. (**A**–**F**) Pipeline for immunophenotyping analysis. (**A**) Representative part of a whole slide scan of a heart section. The cryoablated region is shown with dashed outline. Fluorescence spectra have not been separated in this step. Bar = 500 μm (**B**) Composite view of the area inside the rectangle in panel A after spectral unmixing. Image shows areas of preserved muscle tissue and areas that are mostly scar tissue. Bar = 100 µm. (**C,D**) Representative tissue segmentations performed with an automated algorithm. The segments were used to compartmentalize the cells counted into Muscle and Scar tissue categories. (**E,F**) Nuclear segmentation and phenotyping, respectively. Phenotyping depends on nuclear segmentation, therefore cell portions without a visible nuclear signal are not counted. DAPI = DAPI channel in blue, mCH = mCherry channel in magenta, F4/80 = F4/80 channel in green, Autofl = autofluorescence channel in grey shows the tissue autofluorescence. (**G**,**H**) Number of macrophages and bone marrow derived cells in muscle and scar tissue. Cells numbers presented as a fraction of the total number of cells counted in each region. bmpCtl n = 2 hearts; bmpHet n = 6 hearts; bmpKO n = 5 hearts. No statistically significant differences were found between groups.

Identification of bone marrow derived cells and macrophages was performed on scars and uninjured myocardium regions using multispectral imaging and semi-automated tissue segmentation and phenotyping with anti-mCherry and F4.80 antibodies to identify bone marrow derived cells and macrophages, respectively. Figure 4 shows the mean number of bone marrow derived cells and bone marrow derived macrophages for each donor genotype group. The data show the relative number of bone marrow derived cells (mCh+) is greater in the scar region (1.5–7.0% of all cells) compared with muscle (<1%) 30 days after injury. Moreover, the majority of bone marrow derived cells present in the uninjured myocardium and scar regions are macrophages (mCh+/F4.80+). The number of bone marrow derived macrophages is not significantly different between donor genotype groups in the muscle or scar regions. These data indicate that Cx43 expression does not affect the number of bone marrow derived macrophages present in the scar and surrounding myocardium 30 days after injury. More detailed cell count data are provided in Supplementary Fig. 4.

**Figure 5:**
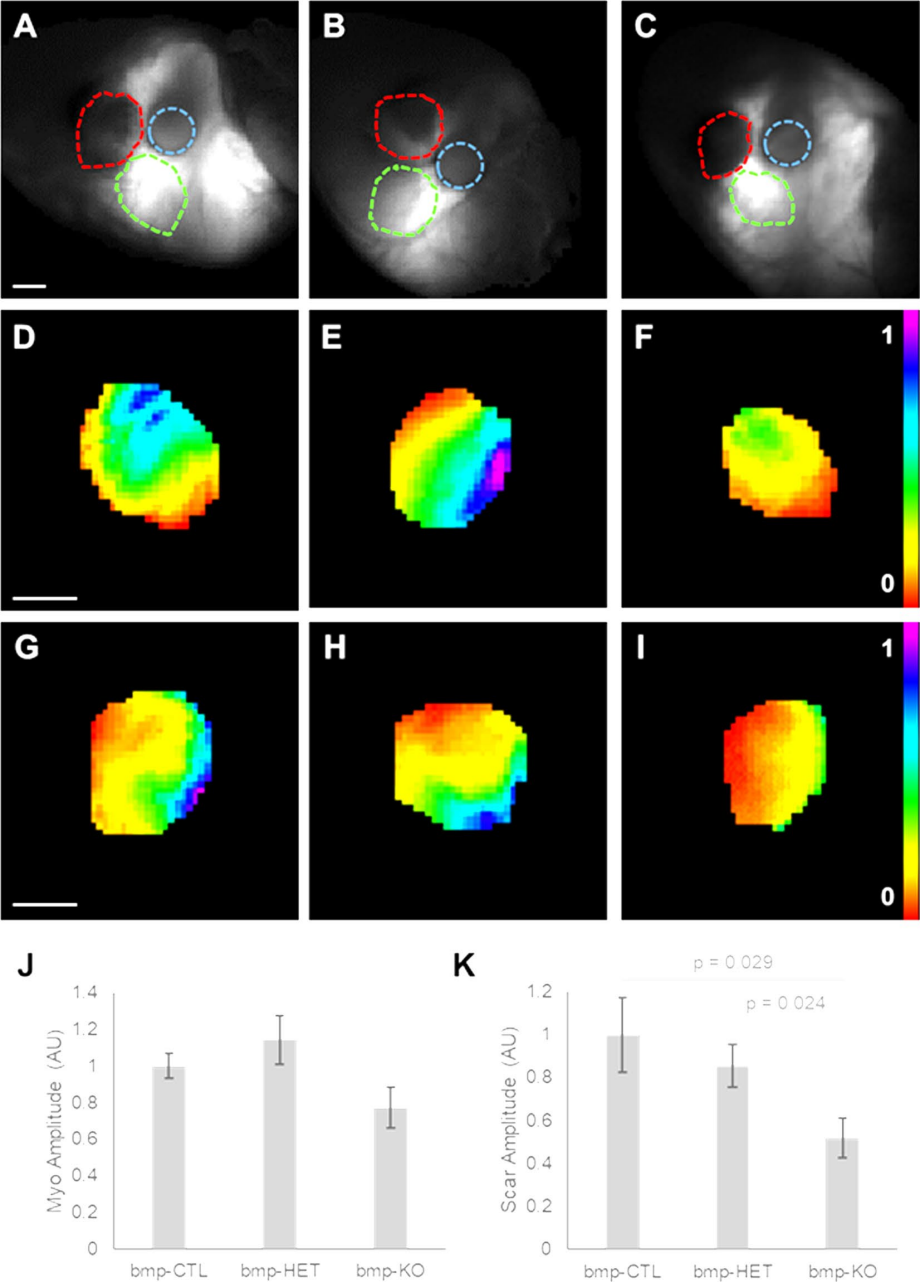
Electrical coupling between uninjured myocardium and scar is mediated in part by Cx43 expression in bone marrow derived cells. (**A–C**) Images from injured hearts transplanted with Cx43^+/+^ (bmp-CTL), Cx43^+/−^ (bmp-HET) and Cx43^−/−^ (bmp-KO) fetal bone marrow progenitors, respectively. Blue line shows the electrode position; green line indicates selected area of uninjured myocardium; red line indicates the scar border. (**D–F**) Maximal voltage maps from uninjured myocardium area shown outlined in green in (**A–C**) respectively. (**G–I**) Maximal voltage maps from scar area shown outlined in red in (**A–C**) respectively. (**J,K**) Average maximal voltage amplitude in the uninjured myocardium and scar areas, respectively. bmp-CTL n = 11; bmp-HET n = 12; bmp-KO n = 10. Bar = 1 mm.

Functional electrical coupling in the scar and uninjured myocardium was evaluated with high resolution optical mapping. Figure 5 shows the spatial changes in maximal membrane voltage in the scar and myocardial regions adjacent to the scar in response to current injection with a suction electrode positioned near the scar. The distance between the center of the electrode and the center of the scar region was on average 2.07 ± 0.05 mm and was not significantly different between groups (bmpCTL 2.04 ± 0.08 mm; bmpHET 1.97 ± 0.08 mm; bmpKO 2.21 ± 0.11 mm). Consistent with the presence of robust electrical coupling in the uninjured myocardium, voltage changes were elicited in the uninjured myocardial regions in response to the current pulses (Fig. 5D–F,J**)**. Voltage amplitudes were highest nearest the electrode and decayed with increasing distance from the electrode in all groups. Average maximal amplitudes appeared to be decreased in the bmpKO group, however it did not reach statistical significance (ANOVA p = 0.07). Voltage changes were also elicited in the scar regions of bmpCTL and bmpHET hearts (Fig. 5G,H). The isopotential maps showed a more pronounced pattern of decay with distance from the electrode compared with the uninjured regions, suggesting reduced electrical coupling in the scar tissue. Measurements in the scar area of the bmpKO group showed a statistically significant reduction in the voltage response compared with the scar regions of the bmpCTL and bmpHET groups, with average voltage values equal to 52.0 ± 9.3% of the control values (Fig. 5I,K). Voltage amplitude at the area of the scar nearest the electrode was decreased and decayed sharply with increasing distance from the electrode, suggesting low electrical coupling between the uninjured myocardium and the scar and within the scar area. These data demonstrate Cx43 expression in bone marrow derived cells is important for the establishment of electrical coupling between the uninjured myocardium and the scar and within the scar tissue itself 30 days after injury.

**Figure 6:**
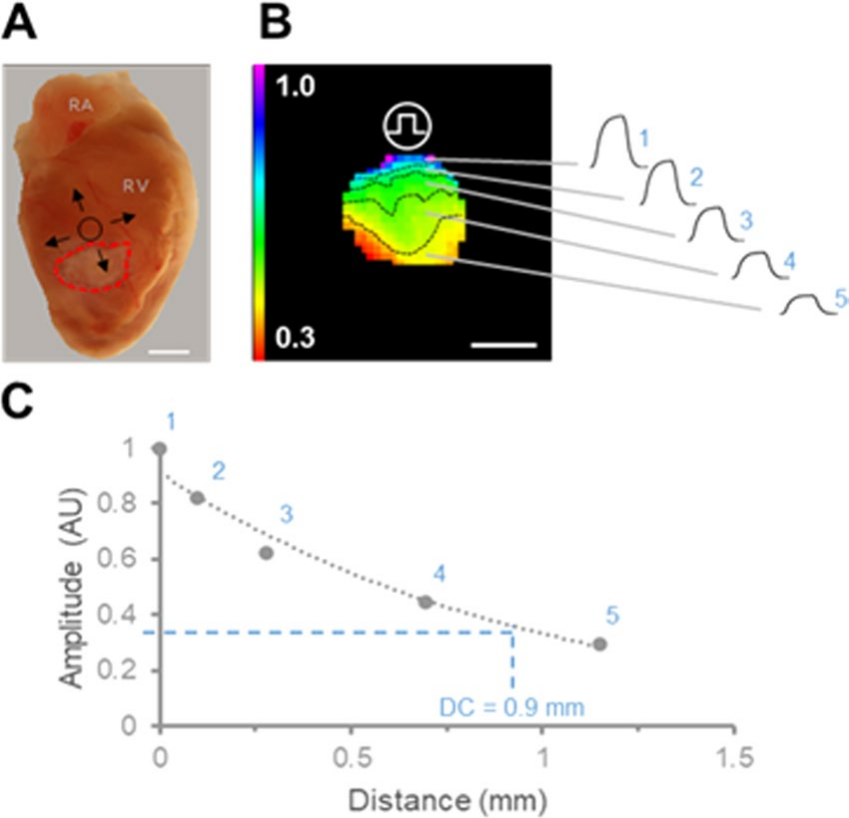
Tissue decay constant calculation. (**A**) Image of heart showing scar area and positioning of the suction electrode. Current is injected adjacent to the scar and spreads passively away from the electrode. Scar area is demarcated in red. (**B**) Left, maximal voltage map obtained from a scar in response to pulsed current. Contour lines divide map into maximal voltage amplitude bins. Each bin is defined to contain the same number of amplitude levels. Approximate electrode location is indicated by the circle. Bar = 1 mm. Right, average voltage pulses obtained from each of the amplitude bins. (**C**) Graph of average maximal voltage versus distance from electrode. Data points 1–5 correspond to the amplitude bins shown in (**B**). Distance is measured radially from the center of the electrode; 0 mm distance corresponds to the edge of the scar closest to the electrode. The decay constant (DC) is calculated from a single exponential fit (dotted line) and corresponds to the distance at which the amplitude equals 37% of the amplitude at 0 mm.

**Figure 7:**
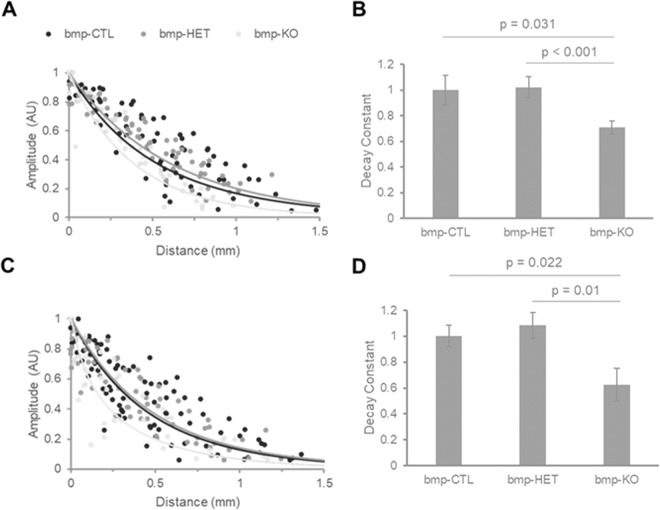
Tissue decay constant. (**A,C**) Graphs of average maximal voltage versus distance from electrode obtained from uninjured myocardium and scar areas, respectively. Animals were transplanted with Cx43^+/+^ (bmp-CTL), Cx43^+/−^ (bmp-HET) and Cx43^−/−^ (bmp-KO) fetal progenitor cells. Individual data points are shown as dots. Solid lines correspond to average single exponential fits for each group. (**B,D**) Average decay constant (DC) for bmp-CTL, bmp-HET and bmp-KO uninjured myocardium and scar areas, respectively. Values are normalized to bmp-CTL average decay constant.

The remodeling of cardiac passive electrical properties has wide implications with regards to arrhythmogenicity and the ability of the tissue to sustain arrhythmic activity. A decay constant parameter was calculated for each heart from the maximal membrane voltage data as illustrated in Fig. 6. Briefly, voltage maps obtained from scar and uninjured myocardial regions were divided into bins, each bin containing an equal range of amplitude levels. The average amplitude for each bin was calculated and graphed against the average distance from the electrode. The decay constant was calculated from a single exponential fit and defined as the distance at which the amplitude equals 37% of the amplitude at the edge of the region closest to the electrode. Figure 7 shows how deletion of Cx43 from bone marrow derived cells alters the decay of voltage as a function of distance from the electrode. Figure 7A,C show individual amplitude measurements and average single exponential fits obtained from uninjured myocardium and scar areas, respectively. Normalized decay constants are shown in Fig. 7B,D. The uninjured myocardium decay constant was 0.57 ± 0.07 mm (n = 9) in the bmpCTL group. The decay constant was not significantly different in the bmpHET group (n = 12), and decreased by 29.2% and 31.5% in the bmpKO group (n = 10) compared with bmpCTL and bmpHET, respectively. Similar changes were identified in the scar region as a consequence of Cx43 deletion. The decay constant in the bmpCTL group was 0.48 ± 0.04 mm (n = 11) and decreased by 37.5% and 45.9% in the bmpKO group (n = 10) compared with bmpCTL and bmpHET, respectively. No significant changes in decay constant were observed in the bmp-HET group (n = 12) compared with control.

## Discussion

Heterocellular electrical interactions between cardiac myocytes and non-myocytes has been a topic of considerable debate since the idea was originally introduced in the 1960s based on experiments performed using primary cultures of neonatal cardiac myocytes and fibroblasts^29,31–43^. Following studies continued to focus in the electrical interactions between myocytes and fibroblasts, partly because fibroblasts constitute the largest cell population in the heart in terms of numbers, and because of their important role in cardiac remodeling in disease states and following cardiac injury. Technological advancement in imaging techniques combined with the increased availability of cell-specific transgenic models has greatly facilitated the study of heterocellular electrical interactions in the intact heart, not only between myocytes and fibroblasts but also between myocytes and other cardiac non-myocyte cell populations including macrophages.

There are now several reports that have identified heterocellular coupling in the intact heart using a variety of imaging and genetic approaches. In 2016 Mahoney *et al*.^15^ demonstrated functional coupling between injured and uninjured cardiac tissue. These studies utilized a cardiac injury model and a fibroblast-specific protein-1 (Fsp1) Cre mouse to target Cx43 expression in cardiac non-myocytes and showed that myocyte-non-myocyte functional coupling is mediated at least in part by Cx43^15^. Fsp1 is expressed in a subset of fibroblast cells and myeloid cells. More recent work from other laboratories have confirmed and extended these findings^2,14,44^. Quinn *et al*.^44^ used the genetically encoded voltage-sensitive fluorescent protein 2.3 expressed in Wilm’s tumor suppressor 1 positive cells to monitor transmembrane potential in cardiac non-myocytes. The detection of action potential-like voltage dependent fluorescence in the border zone of healed cryoinjuries provided direct electrophysiological evidence of heterocellular coupling with myocytes. Another study by Rubart *et al*.^45^ used two-photon laser scanning microscopy in combination with a voltage sensitive dye to simultaneously record voltage signals from myocytes and non-myocytes in infarcted hearts from mice expressing a green fluorescent reporter protein in myocytes. This study found that non-myocytes in the infarct border zone exhibited voltage changes that followed the changes observed in adjacent myocytes. Other studies have explored the electrical interactions between cardiac myocytes and specific non-myocyte cell populations. Hulsmans *et al*.^14^ demonstrated that isolated atrioventricular node macrophages are able to form functional electrical connections and electrically modulate neonatal myocytes in culture. Furthermore, this study showed that in the intact heart optogenetic stimulation of atrioventricular node resident macrophages expressing photoactivatable channelrhodopsin 2 alters atrioventricular node conduction properties, indicating the presence of electrical communication between macrophages and myocytes.

The findings of this paper greatly extend our understanding of the structure and cellular organization of cardiac scar tissue. We manually segmented a SBF-SEM dataset to perform detailed structural analyses of 30-day old RV mouse cardiac scar tissue. The resulting model provided new three-dimensional information regarding cellular volume, cell types, cellular organization, and intercellular contacts. Our data showed approximately 35% of the scar volume is occupied by cells immersed in extracellular matrix. Importantly we observed, and show for the first time, that in spite of the abundant ECM, non-myocyte scar cells form a structural network where individual cells are not isolated. Traditional EM criteria and cell shape information were used to identify the main cell types present in the scar: inflammatory, fibroblastic and endocardial cells. Cellular features observed in EM and immunophenotyping quantification suggest the inflammatory cell population is primarily composed of F4/80 positive macrophages. Finally, segmentation of the cell membranes allowed for quantitative analysis of the cellular contacts established between cells. The presence of intercellular contacts does not indicate the existence junctional channels but it is necessary for the establishment of functional electrical connections between cells. The analysis of intercellular contacts indicated inflammatory cells/macrophages have the largest total intercellular contact area of the three main cell types. Other cell types present in the scar including fibroblasts establish numerous cellular contacts, however the size of these contacts were an order of magnitude smaller than those established between macrophages. These data combined with previous evidence^14,15^ suggest inflammatory cells could have a role in establishing electrophysiological properties and facilitating conduction of electrical activation in scar tissue following injury.

Although very little is known about the electrophysiological role of inflammatory cells, specific inflammatory cell populations and their role in response to cardiac injury has been a topic of intense investigation over the last several years^13^. Inflammatory cells are heterogeneous and the phenotype of these cells changes with time after injury. Following cardiac injury, blood derived monocytes invade the injured region. Initially several inflammatory cell types are present in injured regions including macrophages, neutrophils and mast cells^11,13^. The normal heart contains a network of resident macrophages embedded between the cardiomyocytes. Following myocardial infarction the resident macrophages begin to die and are mostly absent within the first 24 hours. Non-resident activated or pro-inflammatory (M1) macrophages dominate the first three days after infarction, while anti-inflammatory (M2) macrophages are the major cell 5–7 days after infarction. Neutrophils are mostly absent seven days after infarction. Thirty days after injury a mature scar has formed and the majority of immune cells present in the scar are likely macrophages^13^. Extensive remodeling also occurs in the remote uninjured myocardium. There are important differences in the immune response in the remote myocardium compared to the injured region, with changes occurring much slower and continuing to evolve over several months. Unlike the resident macrophages that die shortly after injury in the ischemic regions, in remote regions resident macrophages survive and show increased proliferation. Finally, higher numbers of leukocytes are recruited in the remote myocardium.

In this study, we evaluated the passive conduction properties of 30-day old scars and adjacent uninjured myocardium to assess changes in electrical communication resulting from deletion of Cx43 in blood derived inflammatory cell populations after radiation induced myeloablation. Normal cardiac tissue is highly anisotropic due to directional differences in fiber orientation, cellular architecture, and distribution of gap junctions and membrane ion channels. Moreover degrees of anisotropy vary between regions of the heart. To account for these differences computational studies of cardiac conduction employ bidomain models of cardiac tissue to incorporate more accurate representations of the different parameters affecting the conduction of electrical impulses^46^. Comparably little is known about the properties of cardiac scar tissue, which differs significantly from normal myocardium in multiple aspects including structure and electrophysiological behavior. Most computational studies investigating electrical conduction in infarcted hearts model the intracellular space of scar tissue as insulated from the uninjured myocardium and focus on the effect of changes in the shape and location of the border zone, the degree of transmurality of the scar, and the remodeling of myocyte ion currents in the border zone^47,48^. Although scar tissue is composed of electrically passive cells and extracellular matrix, the assumption that it behaves simply as a barrier to conduction of electrical impulses is contradicted by an increasing number of studies^15,44,45^. Our detailed evaluation of 30-day old RV scar tissue structure also provides evidence that cells in the scar establish physical contacts and form cell networks that could potentially support electrical conduction in the heart.

In our electrophysiological studies current pulses were applied through a suction electrode proximal to the scar while the heart was exposed to the sodium channel blocker flecainide to suppress action potential generation. Hearts were optically mapped and the changes of membrane voltage in space in response to the injected current were determined. The experimental groups were compared based on normalized average maximal voltage amplitudes. The decay of current in space was also analyzed by fitting the changes in membrane voltage with a single exponential function and calculating a decay constant parameter. The electrophysiology data demonstrated the passive conduction properties were significantly altered in the scar and adjacent uninjured myocardium areas of mice transplanted with cells from Cx43 knockout donors compared to mice transplanted with wild type and Cx43 heterozygote donor cells. These findings support the notion that bone marrow derived cells, likely macrophages, significantly contribute to the electrophysiological properties of injured cardiac tissue and that the contribution is at least partially mediated by the expression of Cx43.

The observed membrane potential changes in the scar and uninjured regions are a function of the transmembrane conductance as well as the intercellular, intracellular and extracellular resistivities. Our analysis approach using a single exponential fit to characterize the spread of current in space does not take into consideration possible differential changes in transmembrane conductance. This fact constitutes a limitation, especially concerning the interpretation of the results from the uninjured areas. Furthermore, this study did not evaluate the effect of deletion of Cx43 from bone marrow derived cells on myocyte membrane conductance or extracellular resistance in the intact heart. We believe significant changes in these parameters are unlikely to be present in the chimeric mice, however their contribution to the changes in the passive electrical properties of the tissue cannot be discounted. In contrast to the uninjured myocardium, the cell populations in the scar have comparatively lower transmembrane conductances^28,49^, therefore a significant contribution of transmembrane conductance changes due to Cx43 deletion to the measured passive conduction properties is less likely. Studies have suggested that cardiac conduction may be altered as a result of enhanced mechanosensitive channel activity in response to cardiac injury^50^. In the current study, measurements were obtained in the absence of mechanical contraction. It is possible that mechanosensitive channel activation may contribute to changes in non-junctional conductance in the contracting heart. We also recognize the possibility that other cell types and connexin isoforms may contribute to coupling in within cardiac scars and between myocytes and non-myocytes in the scar. Other parameters that could affect the spread of electrical current in space including wall thickness and fluid in the RV chamber are assumed to be similar between groups. Finally, other mechanisms of intercellular communication that are independent of gap junctions including ephaptic coupling could further enhance the effects of direct electrical coupling provided by gap junctions.

There is now strong evidence supporting heterocellular functional electrical coupling in the intact heart. What remains unknown is the specific cell types that are involved in these electrical interactions. The current study is consistent with prior studies and suggests inflammatory cells are an important cell population that contributes to scar electrophysiology following cardiac injury. More studies are needed to further identify the specific types of inflammatory cells, how functional coupling changes as inflammation progresses during cardiac disease, and to determine the impact of these interactions on the development of cardiac arrhythmias. Studies are also needed to evaluate the functional role of heterocellular coupling in the setting of specific cardiac pathologies.

## Supplementary information

Supplemental Material.

Supplementary Video 1.

Supplementary Video 2.
