# Author Correction: Connexin43 expression in bone marrow derived cells contributes to the electrophysiological properties of cardiac scar tissue

**DOI:** 10.1038/s41598-020-67808-7

**Published:** 2020-07-06

**Authors:** Carolina Vasquez, Valeria Mezzano, Newman Kessler, Freja Swardh, Desiree Ernestad, Vanessa M. Mahoney, John Hanna, Gregory E. Morley

**Affiliations:** Leon H. Charney Division of Cardiology, Department of Medicine, New York University School of Medicine, New York, NY 10016 USA

Correction to: *Scientific Reports* https://doi.org/10.1038/s41598-020-59449-7, published online 13 February 2020

In the original version of this Article, Valeria Mezzano was omitted as a corresponding author. Correspondence about Immunophenotyping and Electron Microscopy should be addressed to Valeria.MezzanoRobinson@nyulangone.org.

As a result, the Author Contributions statement:

“C.V. contributed to experimental design, data analysis and preparation of the manuscript and figures. V.M. contributed to experimental design, performed and supervised E.M. analysis, performed experiments, contributed to data analysis and preparation of manuscript and figures. N.K., F.S., D.E. and J.H. contributed to data analysis. V.M.M. performed experiments and contributed to data analysis. G.E.M. supervised the study, contributed to experimental design, performed experiments, contributed to data analysis and preparation of manuscript and figures.”

now reads,

“C.V. contributed to experimental design, data analysis and preparation of the manuscript and figures. V.M. contributed to experimental design, performed and supervised E.M. analysis, performed experiments, contributed to data analysis and preparation of manuscript and figures. Queries regarding Immunophenotyping and Electron Microscopy should be addressed to V.M. N.K., F.S., D.E. and J.H. contributed to data analysis. V.M.M. performed experiments and contributed to data analysis. G.E.M. supervised the study, contributed to experimental design, performed experiments, contributed to data analysis and preparation of manuscript and figures.”

These errors have now been corrected in the HTML and PDF versions of the original article.

