# Supplementary material for: Connexin43 expression in bone marrow derived cells contributes to the electrophysiological properties of cardiac scar tissue
Source: Sci Rep. 2020 Feb 13;10:2617. doi: 10.1038/s41598-020-59449-7 (PMC7018966; doi:10.1038/s41598-020-59449-7)
Supplement: Supplementary file 1 — Supplemental Material. [file 41598_2020_59449_MOESM1_ESM.pdf]

CONNEXIN43 EXPRESSION IN BONE MARROW DERIVED CELLS CONTRIBUTES TO THE ELECTROPHYSIOLOGICAL PROPERTIES OF CARDIAC SCAR TISSUE

Vasquez C, Mezzano V, Kessler N, Swardh F, Ernestad D,
Mahoney VM, Hanna J, Morley GE

SUPPLEMENTAL MATERIAL

Supplementary Video 1. Animation of a representative slice of the SBF-SEM stack. Video shows the magnitude of the X-Y area that was analyzed and zooms in to reveal the highest working magnification.

Supplementary Video 2. Three-dimensional models of a subset of cells segmented in the SBF-SEM dataset together with the EM images from which they were produced. EM images are slowly removed to show the cell models. The cell models and then rotated to visualize the cells from different angles. Finally, three-dimensional models of intercellular contacts are shown in dark blue. The cell models are removed to display the density of connections in relation to space.

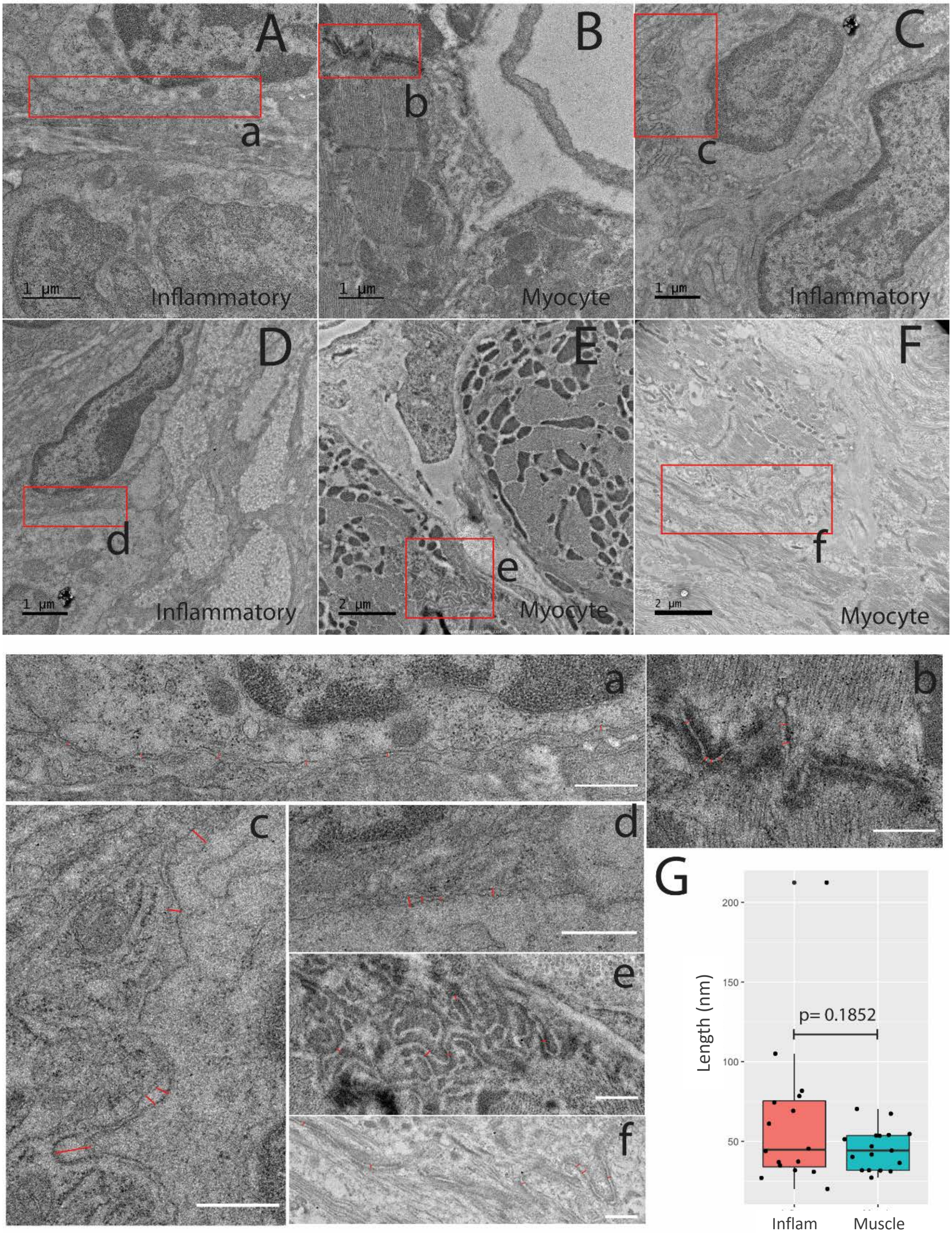

Supplementary Figure 1. Intermembrane distance between non-myocytes in scar tissue. TEM images of cell to cell contacts in 30-day old scars at low (**A, B, C, D, E, F**) and high (**a, b, c, d, e, f**) magnification. Intermembrane distances were sampled between inflammatory cells and at the intercalated discs of cardiac myocytes. Red lines show the regions from where measurements were taken. **G.** Box plots showing comparison of distances between inflammatory cells (Inflam) and between cardiac myocytes (Muscle).

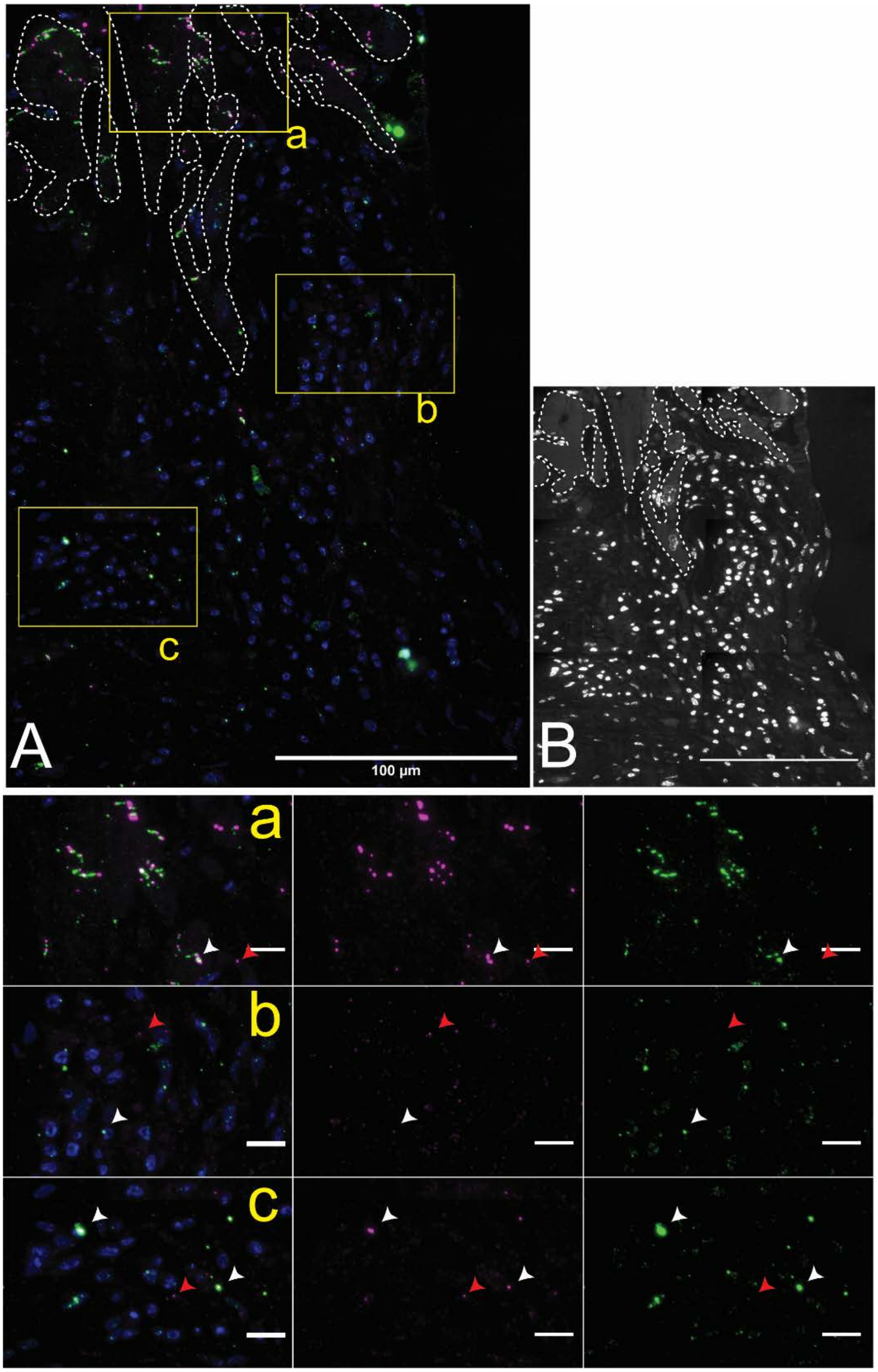

Supplementary Figure 2. Widefield immunofluorescence microscopy of LR-White embedded scar section stained using primary antibodies against Cx43 and pan-Cadherin. A. Merged montage of the imaged area. White outline shows myocyte borders. **B.** Grayscale image of area shown in A. **a-c** Higher magnification images of the area inside the insets in A. White arrowheads indicate regions where Cx43 (magenta) and pan-Cadherin (green) colocalize, red arrowheads show spots where Cx43 is present without pan-cadherin immunoreactivity. Scale bars = 100 μm in A and B, 10 μm in a-c.

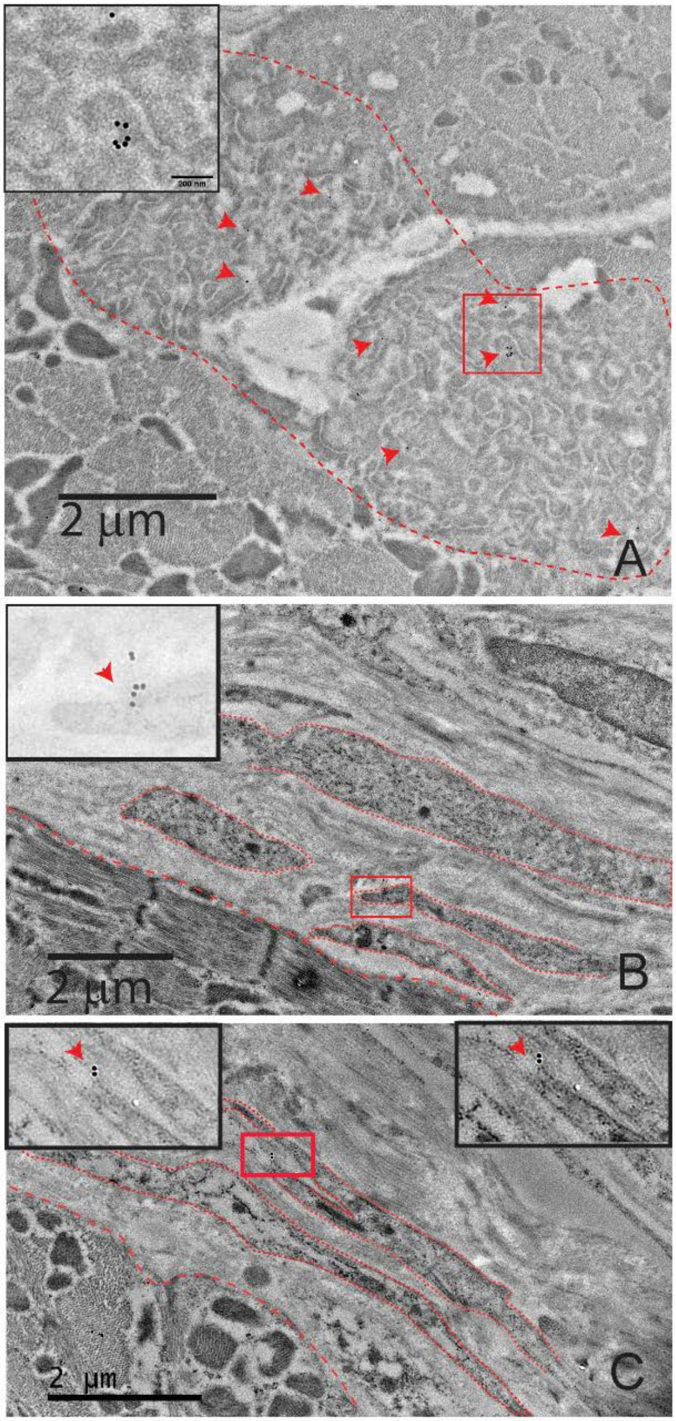

Supplementary Figure 3. Non-myocyte cells have positive immunoreactivity to Cx43 in 30-day old scars. A. Cross section of an intercalated disc (outlined with dashed red line) with positive immunoreactivity to Cx43 (immunogold beads, arrowheads). Inset shows higher magnification of the area inside the red square. **B-C.** Border region of scar showing part of a cardiac myocyte (dashed line) and non-myocyte cells (dotted lines). Inset shows a low contrast image for improved gold bead visualization.

A

MouseID	Fields	Total	Mean	SEM
BMT14.10	16	9513	594.56	55.89
BMT14.11	13	10319	793.77	46.69
BMT14.14	12	8101	675.08	54.75
BMT14.17	12	7356	613.00	80.92
BMT14.18	12	7579	631.58	76.05
BMT14.19	16	10594	662.12	57.83
BMT14.20	11	7101	645.55	76.07
BMT14.3	11	7248	658.91	45.66
BMT14.4	16	12039	752.44	63.26
BMT14.6	17	11491	675.94	40.82
BMT14.7	10	7199	719.90	84.66
BMT14.8	15	10837	722.47	41.02
BMT14.9	13	9185	706.54	75.74

B

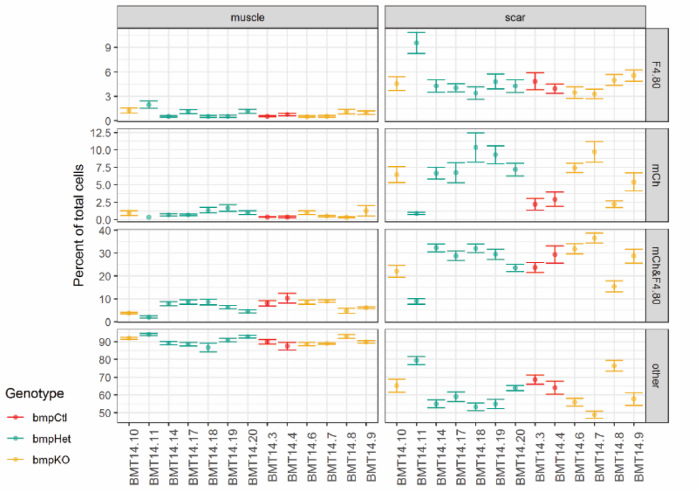

Supplementary Figure 4. Cell Counts. A. Table summarizing the number of fields acquired for analysis, and the total, mean and standard error of the mean (SEM) of the number of cells counted per mouse per field. **B.** Cell counts for each cell phenotype shown as a percent of the total number of cells counted. Mean and standard error of the mean are shown for each individual mouse.
